# Effect of overall lifestyle on the all-cause mortality and cardiovascular disease death in dyslipidemia patients with or without lipid-lowering therapy: a cohort study

**DOI:** 10.1186/s12872-023-03450-1

**Published:** 2023-09-04

**Authors:** Qian Wang, Dong Pang, Hui Wang

**Affiliations:** 1 Changsheng Community Health Service Station, Jinan Hospital, Jinan, 250014 P.R. China; 2 Department of General Practice, Jinan Hospital, Jinan, 250014 P.R. China; 3 Department of Emergency, Jinan Hospital, No.63-1 Lishan Road, Lixia District, Jinan, 250014 P.R. China

**Keywords:** Overall lifestyle, Dyslipidemia, All-cause mortality, CVD death

## Abstract

**Background:**

Lifestyle adjustment has been reported as one of the interventions for dyslipidemia. This study aimed to explore the effect of overall lifestyle on the risk of all-cause mortality and cardiovascular disease (CVD) death in dyslipidemia patients with or without lipid-lowering therapy.

**Methods:**

This was a retrospective cohort study, and data were extracted from the National Health and Nutrition Examination Survey (NHANES). Overall lifestyle was assessed based on Mediterranean diet score, physical activity, smoking status, sleep duration, and body mass index (BMI). Multivariate Cox regression model was used to explore the effect of overall lifestyle score on the risk of all-cause mortality and CVD death. Results were shown as hazard ratio (HR), with 95% confidence interval (CI).

**Results:**

A total of 11,549 dyslipidemia patients were finally included in this study. The results showed that optimal overall lifestyle was associated with the decreased risk of all-cause mortality (HR = 0.47, 95%CI: 0.34–0.64) and CVD death (HR = 0.45, 95%CI: 0.22–0.94) in patients without lipid-lowering therapy. The similar results were found in patients with lipid-lowering therapy (all-cause mortality: HR = 0.45, 95%CI: 0.33–0.62; CVD death: HR = 0.38, 95%CI: 0.23–0.63).

**Conclusions:**

A favorable overall lifestyle may have great benefits to improve the prognosis of dyslipidemia, highlighting the importance of overall lifestyle adjustment for dyslipidemia patients.

**Supplementary Information:**

The online version contains supplementary material available at 10.1186/s12872-023-03450-1.

## Background

Dyslipidemia is defined as an increase in the plasma concentrations of total cholesterol (TC), low density lipoprotein-cholesterol (LDL-C) or triglycerides (TG), or a low plasma concentration of high-density lipoprotein cholesterol (HDL-C), or a combination of these features [1]. The prevalence of dyslipidemia is approximately 18% in the United States, and has been a main risk factor for cardiovascular disease (CVD) [1]. Dyslipidemia accounts for about 4.40 million global death and 98.62 million disabilities in 2019, which causes a big burden to medical system [1].

Lipid-lowering medications have been used for the treatment of dyslipidemia; however, side effect of lipid-lowering drugs is a problem that cannot be ignored [2]. The American Heart Association (AHA) recommends a healthy lifestyle across life course and judicious use of lipid-lowering drugs depending on the patients’ risk status [3]. Previous studies have reported that the single lifestyle factor (diet or fitness) combined with statin drugs were associated with the reduced level of cholesterol and decreased risk of mortality [4, 5]. It was known that human lifestyle was overall rather than single [6]. A study by Booth et al. have found that a higher number of healthy lifestyle factors was associated with the lower incidence of atherosclerotic cardiovascular diseases and lower risk of all-cause mortality in dyslipidemia patients with statin therapy [7]. However, this finding needed to be further verified.

In addition, evidence showed that the prescription rate for statins is low in dyslipidemia patients, and 52.3% of dyslipidemia patients do not receive statin therapy [8]. Therefore, adjusting lifestyle is recommended as the primary method for dyslipidemia patients without lipid-lowering therapy [3]. Trautwein et al. have found that a plant-based diet was conducive to lowering LDL-C level and decreasing the risk of CVD in dyslipidemia patients [9]. However, the effect of overall lifestyle on the prognosis of dyslipidemia patients without lipid-lowering therapy has not been reported.

In this study, we aimed to further explore the association between overall lifestyle and the risk of CVD death and all-cause mortality in dyslipidemia patients with lipid-lowering therapy. Also, we explored the effect of overall lifestyle on the risk of CVD death and all-cause mortality in dyslipidemia patients without lipid-lowering therapy.

## Methods

### Study design and data source

This was a retrospective cohort study based on the National Health and Nutrition Examination Survey (NHANES) (https://www.cdc.gov/nchs/nhanes/index.htm). NHANES was a program of the National Center for Health Statistics (NCHS) of the Centers for Disease Control and Prevention (CDC), and examined a nationally representative sample of about 5,000 people each year [10]. NHANES combined interviews and physical examinations. Interviews consisted of demographic, socioeconomic, dietary, and health-related questions. Examination section included medical, dental, physiological measurements, and laboratory tests. Seven cycles of NHANES from 2005 to 2018 (2005–2006, 2007–2008, 2009–2010, 2011–2012, 2013–2014, 2015–2016, and 2017–2018) were downloaded. The protocols from NHANES have all been approved by the National Center for Health Statistics Research Ethics Review Board (ERB) [11]. The requirement of ethical approval for this was waived by the Institutional Review Board of Jinan Hospital, because the data was accessed from NHANES (a publicly available database). Written informed consent was not required as this study was based on publicly available data. All methods were performed in accordance with the relevant guidelines and regulations.

### Study population

Participants meeting the following criteria were included: (1) age ≥ 18 years old; (2) with dyslipidemia: TC ≥ 200 mg/dL (5.2 mmol/L), TG ≥ 150 mg/dL (1.7 mmol/L), LDL-C ≥ 130 mg/dL (3.4 mmol/L), HDL-C ≤ 40 mg/dL (1.0 mmol/L) [12], self-reported hypercholesterolemia, or undergoing lipid-lowering therapy; and (3) with data on lifestyle factors. Participants meeting the following criteria were excluded: (1) missing data on survival.

### Lipid-lowering therapy

Participants with self-reported use of lipid-lowering drugs and cholesterol-lowering treatments were determined to receive lipid-lowering therapy. When be asked “In the past 30 days, have you used or taken medication for which a prescription is needed? Do not include prescription vitamins or minerals you may have already told me about”, participants answering “Yes” were then asked for drug name. Participants reporting 3-Hydroxy-3-methyl glutaryl coenzyme A (HMG-COA) reductase inhibitors (statins), miscellaneous antihyperlipidemic agents, fibric acid derivatives, bile acid sequestrants, cholesterol absorption inhibitors, antihyperlipidemic combinations, and PCSK9 inhibitors were regarded as taking lipid-lowering drugs.

### Overall lifestyle assessment

Overall lifestyle was assessed based on the following five lifestyle factors: Mediterranean diet score, physical activity level, smoking status, sleep duration, and BMI [6, 7]. Each category was divided into poor, intermediate, and optimal, which scored 0, 1, and 2, respectively. Overall lifestyle score was the sum of the individual score of all 5 lifestyle factors, and divided into poor (0–3 points), intermediate (4–6 points), and optimal (7–10 points).

Mediterranean diet score included 9 components, and sex-specific medians were used as the cutoff to dichotomize the food groups [13, 14]. One point was assigned to the consumption above the median of presumed beneficial foods (legumes, cereals, fruits and nuts, high vegetables, fish, monounsaturated fatty acids-to-saturated fatty acids ratio) and the consumption below the median of presumed detrimental foods (meat and meat products, and dairy products). For alcohol, one point was assigned to men who consumed 10–25 g/day and to women who consumed 5–15 g/day. The total score of Mediterranean diet was 18, and divided into poor (0–6 points), intermediate (7–11 points), and optimal (12–18 points).

Physical activity was divided into high level (scored 2 points) and low level (scored 1 points). For work or sports/fitness/recreational activities, participant did one of these activities at vigorous-intensity, which caused a large increase in breathing or heart rate for at least 10 min continuously, was considered as high level of physical activity. For work, usual way to and from places, and sports/fitness/recreational activities, participants did two of these activities at moderate-intensity, which caused a small increase in breathing or heart rate for at least 10 min continuously, was considered as high level of physical activity. Otherwise, participants were considered at low level of physical activity.

For smoking status, when be asked “Do you smoked at least 100 cigarettes in life” and “Do you now smoke cigarettes”, someone who answered “No” for the first question was classified as “never smoker”; someone who answered “Yes” for the first question and “No” for the second question was classified as “former smoker”; someone who answered “Yes” for both the first and the second question was classified as “current smoker” [15]. Smoking status was divided into poor (current smoking), intermediate (former smoking), and optimal (never smoking).

Sleep duration was obtained from the question “How much sleep do you usually get at night on weekdays or workdays”, and divided into poor (< 5 h/day or > 10 h/day), intermediate (5-5.9 h/day or 8.1–10 h/day), and optimal (6–8 h/day) [16].

BMI was calculated as body weight (kg)/height (m^2^), and divided into underweight (BMI < 18.5 kg/m^2^), normal weight (18.5 kg/m^2^ ≤ BMI < 25 kg/m^2^), overweight (25 kg/m^2^ ≤ BMI < 30 kg/m^2^), and obesity (BMI ≥ 30 kg/m^2^) [17]. This category was divided into optimal (normal weight), intermediate (underweight or overweight), and poor (obesity).

### Outcomes

Outcomes were all-cause mortality and CVD death. All-cause mortality was defined as death from any cause, and CVD death was defined as death from heart diseases and cerebrovascular diseases [18]. The follow-up was terminated when the included patients died.

### Covariates of interest

Data of included patients were obtained, including age, gender, race, education level, poverty income ratio (PIR), triglyceride, LDL-C, TC, HDL-C, history of CVD, family history of CVD, hypertension, and diabetes.

### Statistical analysis

Measurement data in normal distribution were expressed as mean ± standard deviation (mean ± SD), and differences were compared using t-test (between two groups) or variance analysis (more than two groups). Measurement data in non-normal distribution were expressed as median [M (Q1, Q3)], and differences between groups were compared using rank sum test. Counting data were expressed as number (n) and percentage (%), and differences in groups were compared using chi-square test. Missing data were processed using multiple imputation method, and sensitivity analysis was performed before and after the interpolation.

Univariate Cox regression model was used to select the potential confounders, and the selected confounders were then adjusted in the multivariate Cox regression model to explore the effect of overall lifestyle score on the all-cause mortality and CVD death. Given that BMI could be an intermediate factor between behavioral factors and health outcomes [19], sensitivity analysis which excluded BMI from the overall lifestyle score and included it as a covariate was performed to explore the effect of overall lifestyle score on the all-cause mortality and CVD death. Subgroup analysis was performed based on age. Statistical analysis was performed using SAS 9.4 (SAS Institute Inc., Cary, NC, USA) and R (version 4.2.1, R Foundation for Statistical Computing, Vienna, Austria). *P* < 0.05 was considered as statistical difference.

## Results

### Selection and baseline information of patients

**Fig. 1:**
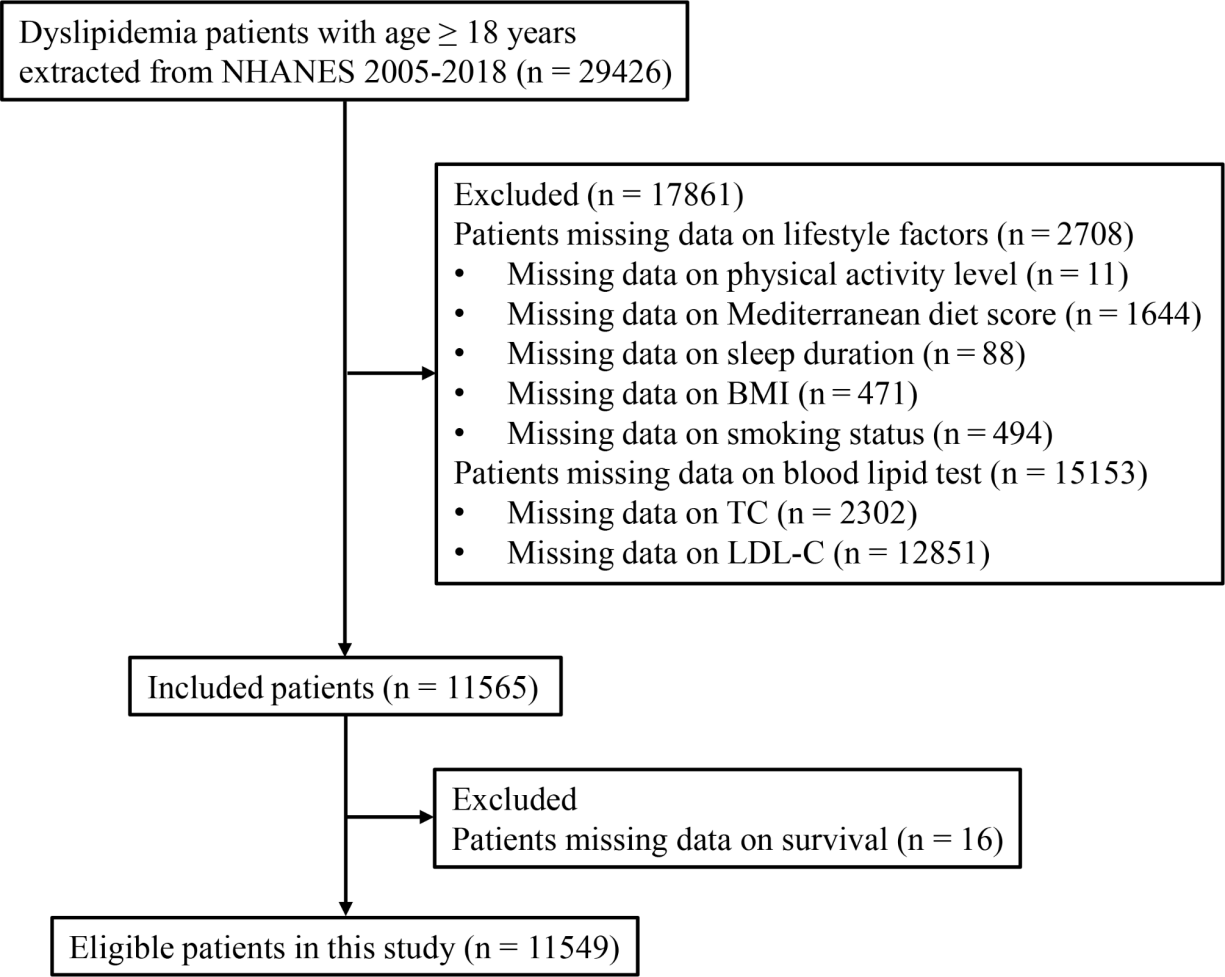
The flowchart of patient section

A total of 29,426 dyslipidemia patients with age ≥ 18 years were extracted from 2005 to 2018 NHANES database. Of these, we excluded 2,708 patients missing data on lifestyle factors [physical activity level (n = 11); Mediterranean diet score (n = 1644); sleep duration (n = 88), BMI (n = 471), and smoking status (n = 494)] and 15,153 patients missing data on blood lipid test [TC (n = 2302) and LDL-C (n = 12,851)]. In the remaining 11,565 patients, 16 patients were excluded due to missing data on survival. Finally, 11,549 patients were included in this study for analysis (Fig. 1).

In the included patients, there were 1,109 patients in the poor overall lifestyle group, 7,292 patients in the intermediate overall lifestyle group, and 3,148 patients in the optimal overall lifestyle group. Sensitivity analysis showed that estimates were not significantly different before versus after imputation for missing data (all *P* > 0.05) (Additional file table S1). The mean age of included patients was 53.54 ± 17.08 years, and there were 50.51% of male (n = 5833). In addition, there were 32.15% of patients with lipid-lowering therapy (n = 3713) and 67.85% of patients without lipid-lowering therapy (n = 7836) (Table 1). The characteristics of patients without lipid-lowering therapy were shown in Additional file table S2, and the characteristics of patients with lipid-lowering therapy were shown in Additional file table S3.

**Table 1:** Characteristics of included patients (total patients)

Variables	Total (n = 11,549)	Overall lifestyle score			Statistics	*P*
		Poor (n = 1109)	Intermediate (n = 7292)	Optimal (n = 3148)		
Age, year, Mean ± SD	53.54 ± 17.08	53.33 ± 15.60	54.07 ± 17.20	52.39 ± 17.24	F = 10.758	< 0.001
Gender, n (%)					χ^2^ = 0.878	0.645
Male	5833 (50.51)	566 (51.04)	3699 (50.73)	1568 (49.81)		
Female	5716 (49.49)	543 (48.96)	3593 (49.27)	1580 (50.19)		
Race, n (%)					χ^2^ = 413.636	< 0.001
Mexican American	1816 (15.72)	132 (11.90)	1197 (16.42)	487 (15.47)		
Other Hispanic	1208 (10.46)	105 (9.47)	779 (10.68)	324 (10.29)		
Non-Hispanic white	5123 (44.36)	495 (44.63)	3291 (45.13)	1337 (42.47)		
Non-Hispanic black	2165 (18.75)	305 (27.50)	1460 (20.02)	400 (12.71)		
Other race-including multi-racial	1237 (10.71)	72 (6.49)	565 (7.75)	600 (19.06)		
Education level, n (%)					χ^2^ = 319.836	< 0.001
Less than 9th grade	1380 (11.95)	146 (13.17)	936 (12.84)	298 (9.47)		
9-11th grade (including 12th grade with no diploma)	1733 (15.01)	258 (23.26)	1168 (16.02)	307 (9.75)		
High school graduate/ GED or equivalent	2722 (23.57)	322 (29.04)	1781 (24.42)	619 (19.66)		
More than high school	5714 (49.48)	383 (34.54)	3407 (46.72)	1924 (61.12)		
PIR, %, M (Q_1_, Q_3_)	2.14 (1.16, 4.08)	1.43 (0.92, 2.74)	2.04 (1.12, 4.00)	3.01 (1.53, 5.00)	χ^2^ = 428.211	< 0.001^#^
Triglyceride, mmol/L, M (Q_1_, Q_3_)	1.36 (0.96, 1.94)	1.57 (1.08, 2.12)	1.42 (1.01, 2.02)	1.19 (0.84, 1.72)	χ^2^ = 294.536	< 0.001^#^
LDL-C, mmol/L, M (Q_1_, Q_3_)	3.15 ± 0.98	3.05 (2.33, 3.70)	3.16 (2.43, 3.75)	3.28 (2.61, 3.78)	χ^2^ = 35.813	< 0.001^#^
TC, mmol/L, Mean ± SD	5.24 ± 1.11	5.09 ± 1.14	5.21 ± 1.12	5.34 ± 1.06	F = 26.139	< 0.001
HDL-C, mmol/L, Mean ± SD	1.38 ± 0.44	1.24 ± 0.41	1.34 ± 0.43	1.50 ± 0.47	F = 202.652	< 0.001
History of CVD, n (%)					χ^2^ = 177.962	< 0.001
Yes	1676 (14.51)	277 (24.98)	1115 (15.29)	284 (9.02)		
No	9873 (85.49)	832 (75.02)	6177 (84.71)	2864 (90.98)		
Family history of CVD, n (%)					χ^2^ = 81.936	< 0.001
Yes	1540 (13.33)	226 (20.38)	999 (13.70)	315 (10.01)		
No	9618 (83.28)	846 (76.28)	6034 (82.75)	2738 (86.98)		
Unknown	391 (3.39)	37 (3.34)	259 (3.55)	95 (3.02)		
Hypertension, n (%)					χ^2^ = 263.906	< 0.001
Yes	6471 (56.03)	735 (66.28)	4345 (59.59)	1391 (44.19)		
No	5078 (43.97)	374 (33.72)	2947 (40.41)	1757 (55.81)		
Diabetes					χ^2^ = 237.086	< 0.001
Yes	3009 (26.05)	421 (37.96)	2057 (28.21)	531 (16.87)		
No	8540 (73.95)	688 (62.04)	5235 (71.79)	2617 (83.13)		
BMI, kg/m^2^, Mean ± SD	29.71 ± 6.75	34.13 ± 7.01	30.81 ± 6.79	25.63 ± 4.07	F = 1082.654	< 0.001
Smoking status, n (%)					χ^2^ = 3070.639	< 0.001
Poor	2304 (19.95)	745 (67.18)	1519 (20.83)	40 (1.27)		
Intermediate	3126 (27.07)	280 (25.25)	2365 (32.43)	481 (15.28)		
Optimal	6119 (52.98)	84 (7.57)	3408 (46.74)	2627 (83.45)		
Sleep duration, n (%)					χ^2^ = 2825.153	< 0.001
Poor	817 (7.07)	434 (39.13)	379 (5.20)	4 (0.13)		
Intermediate	2498 (21.63)	415 (37.42)	1821 (24.97)	262 (8.32)		
Optimal	8234 (71.30)	260 (23.44)	5092 (69.83)	2882 (91.55)		
BMI category, n (%)					χ^2^ = 2935.314	< 0.001
Poor	4716 (40.83)	879 (79.26)	3596 (49.31)	241 (7.66)		
Intermediate	4185 (36.24)	195 (17.58)	2651 (36.35)	1339 (42.53)		
Optimal	2648 (22.93)	35 (3.16)	1045 (14.33)	1568 (49.81)		
Mediterranean diet score, n (%)					χ^2^ = 1918.314	< 0.001
Poor	8856 (76.68)	1061 (95.67)	5809 (79.66)	1332 (42.31)		
Intermediate	2629 (22.76)	48 (4.33)	1475 (20.23)	1733 (55.05)		
Optimal	64 (0.55)	0 (0.00)	8 (0.11)	83 (2.64)		
Physical activity level, n (%)					χ^2^ = 1261.554	< 0.001
Low	6250 (54.12)	931 (83.95)	4385 (60.13)	934 (29.67)		
High	5299 (45.88)	178 (16.05)	2907 (39.87)	2214 (70.33)		
Lipid-lowering therapy, n (%)					χ^2^ = 82.302	< 0.001
No	7836 (67.85)	685 (61.77)	4823 (66.14)	2328 (73.95)		
Yes	3713 (32.15)	424 (38.23)	2469 (33.86)	820 (26.05)		
Follow-up time, month, M (Q_1_, Q_3_)	89.00 (49.00, 130.00)	81.00 (41.00, 123.00)	89.00 (49.00, 130.00)	93.00 (55.00, 130.00)	χ^2^ = 30.408	< 0.001^#^
CVD death, n (%)					χ^2^ = 65.176	< 0.001
Survival	10,133 (87.74)	917 (82.69)	6345 (87.01)	2871 (91.20)		
CVD death	459 (3.97)	61 (5.50)	311 (4.26)	87 (2.76)		
Other death	957 (8.29)	131 (11.81)	636 (8.72)	190 (6.04)		
All-cause mortality, n (%)					χ^2^ = 64.950	< 0.001
Survival	10,133 (87.74)	917 (82.69)	6345 (87.01)	2871 (91.20)		
Death	1416 (12.26)	192 (17.31)	947 (12.99)	277 (8.80)		

Abbreviation: Mean ± SD, mean ± standard deviation; GED, General Educational Development; PIR, poverty-income ratio; LDL-C, low-density lipoprotein cholesterol; TC, total cholesterol; HDL-C, high-density lipoprotein cholesterol; CVD, cardiovascular disease; BMI, body mass index

χ^2^: chi-square test; F: variance analysis; #: rank sum test

### Effect of overall lifestyle on the risk of all-cause mortality and CVD death in dyslipidemia patients with or without lipid-lowering therapy

Table 2 shows that age, race, education level, PIR, HDL-C, family history of CVD, history of CVD, hypertension, and diabetes were covariates for all-cause mortality in patients without lipid-lowering therapy. Age, race, education level, PIR, HDL-C, history of CVD, hypertension, and diabetes were covariates for CVD death in patients without lipid-lowering therapy. Age, race, education level, PIR, LDL-C, TC, history of CVD, hypertension, and diabetes were covariates for all-cause mortality in patients with lipid-lowering therapy. Age, education level, PIR, LDL-C, TC, history of CVD, hypertension, and diabetes were covariates for CVD death in patients with lipid-lowering therapy.

**Table 2:** Covariates for all-cause mortality and CVD death in dyslipidemia patients with or without lipid-lowering therapy

Characteristics	patients without lipid-lowering therapy				patients with lipid-lowering therapy			
	All-cause mortality		CVD death		All-cause mortality		CVD death	
	HR (95%CI)	*P*	HR (95%CI)	*P*	HR (95%CI)	*P*	HR (95%CI)	*P*
Age, year	1.10 (1.09–1.11)	< 0.001	1.14 (1.12–1.16)	< 0.001	1.11 (1.09–1.12)	< 0.001	1.11 (1.09–1.14)	< 0.001
Gender								
Male	Ref		Ref		Ref		Ref	
Female	1.14 (0.93–1.39)	0.197	1.28 (0.93–1.77)	0.136	0.88 (0.75–1.02)	0.091	0.81 (0.62–1.06)	0.124
Race								
Mexican American	Ref		Ref		Ref		Ref	
Other Hispanic	0.89 (0.53–1.50)	0.672	0.55 (0.22–1.35)	0.191	1.34 (0.80–2.24)	0.266	1.85 (0.81–4.22)	0.143
Non-Hispanic white	2.81 (1.94–4.05)	< 0.001	1.90 (1.03–3.51)	0.040	1.54 (1.10–2.14)	0.011	1.65 (0.97–2.82)	0.067
Non-Hispanic black	2.60 (1.80–3.74)	< 0.001	2.50 (1.33–4.72)	0.005	1.42 (0.98–2.06)	0.067	1.64 (0.90–2.99)	0.103
Other race-including multi-racial	1.32 (0.76–2.28)	0.326	1.72 (0.65–4.60)	0.278	0.78 (0.41–1.49)	0.450	0.85 (0.30–2.45)	0.766
Education level								
Less than 9th grade	Ref		Ref		Ref		Ref	
9-11th grade (including 12th grade with no diploma)	0.79 (0.58–1.07)	0.122	0.67 (0.43–1.05)	0.080	0.86 (0.69–1.08)	0.190	0.85 (0.57–1.29)	0.449
High school graduate/ GED or equivalent	0.65 (0.46–0.91)	0.011	0.39 (0.22–0.68)	0.001	0.63 (0.47–0.83)	0.001	0.77 (0.49–1.21)	0.260
More than high school	0.44 (0.33–0.60)	< 0.001	0.33 (0.21–0.53)	< 0.001	0.40 (0.30–0.53)	< 0.001	0.41 (0.27–0.61)	< 0.001
PIR, %	0.80 (0.76–0.85)	< 0.001	0.79 (0.71–0.87)	< 0.001	0.75 (0.69–0.80)	< 0.001	0.74 (0.67–0.81)	< 0.001
Triglyceride, mmol/L	1.01 (0.89–1.14)	0.914	1.10 (0.90–1.34)	0.373	0.90 (0.81–1.01)	0.069	0.92 (0.77–1.10)	0.359
LDL-C, mmol/L	0.90 (0.78–1.04)	0.163	0.96 (0.77–1.19)	0.719	0.71 (0.62–0.81)	< 0.001	0.69 (0.55–0.88)	0.002
TC, mmol/L	1.00 (0.88–1.13)	0.999	1.11 (0.95–1.31)	0.198	0.77 (0.69–0.85)	< 0.001	0.72 (0.59–0.88)	0.002
HDL-C, mmol/L	1.37 (1.00-1.86)	0.047	1.54 (1.13–2.08)	0.005	1.11 (0.86–1.42)	0.420	0.82 (0.52–1.27)	0.374
Family history of CVD								
Yes	Ref		Ref		Ref		Ref	
No	0.67 (0.53–0.86)	0.001	0.87 (0.50–1.51)	0.621	0.92 (0.68–1.25)	0.605	0.75 (0.46–1.23)	0.255
Unknown	1.26 (0.73–2.18)	0.401	1.70 (0.56–5.18)	0.347	1.50 (0.91–2.47)	0.116	1.35 (0.69–2.63)	0.382
History of CVD								
Yes	Ref		Ref		Ref		Ref	
No	0.14 (0.12–0.18)	< 0.001	0.09 (0.06–0.13)	< 0.001	0.32 (0.27–0.39)	< 0.001	0.27 (0.20–0.35)	< 0.001
Hypertension								
Yes	Ref		Ref		Ref		Ref	
No	0.29 (0.22–0.38)	< 0.001	0.17 (0.10–0.28)	< 0.001	0.52 (0.41–0.67)	< 0.001	0.36 (0.24–0.53)	< 0.001
Diabetes								
Yes	Ref		Ref		Ref		Ref	
No	0.27 (0.21–0.35)	< 0.001	0.21 (0.15–0.3)	< 0.001	0.64 (0.53–0.76)	< 0.001	0.64 (0.47–0.85)	0.003

Abbreviation: CVD, cardiovascular disease; HR, hazard ratio; CI, confidence interval; GED, General Educational Development; PIR, poverty-income ratio; LDL-C, low-density lipoprotein cholesterol; TC, total cholesterol; HDL-C, high-density lipoprotein cholesterol

After adjusting these covariates, we found that better overall lifestyle was associated with the lower risk of all-cause mortality (intermediate: HR = 0.68, 95%CI: 0.52–0.90; optimal: HR = 0.47, 95%CI: 0.34–0.64) and CVD death (optimal: HR = 0.45, 95%CI: 0.22–0.94) than the poor overall lifestyle in patients without lipid-lowering therapy. We also found the significant association between better overall lifestyle and lower risk of all-cause mortality (intermediate: HR = 0.55, 95%CI: 0.42–0.73; optimal: HR = 0.45, 95%CI: 0.33–0.62) and CVD death (intermediate: HR = 0.46, 95%CI: 0.32–0.68; optimal: HR = 0.38, 95%CI: 0.23–0.63) in patients with lipid-lowering therapy (Table 3). Additional file table S4 shows the similar association between lifestyle and the risk of all-cause mortality and CVD death after removing BMI from the overall lifestyle score.

**Table 3:** Association between lifestyle score level and all-cause mortality or CVD death in dyslipidemia patients with or without lipid-lowering therapy

Model	Overall lifestyle score	Patients without lipid-lowering therapy^#^				Patients with lipid-lowering therapy^*^			
		All-cause mortality		CVD death		All-cause mortality		CVD death	
		HR (95%CI)	*P*	HR (95%CI)	*P*	HR (95%CI)	*P*	HR (95%CI)	*P*
Model 1	Poor	Ref		Ref		Ref		Ref	
	Intermediate	0.68 (0.52–0.88)	0.003	0.73 (0.37–1.45)	0.371	0.68 (0.51–0.9)	0.008	0.54 (0.35–0.83)	0.005
	Optimal	0.43 (0.32–0.56)	< 0.001	0.47 (0.24–0.93)	0.031	0.48 (0.35–0.66)	< 0.001	0.38 (0.23–0.63)	< 0.001
Model 2	Poor	Ref		Ref		Ref		Ref	
	Intermediate	0.68 (0.52–0.90)	0.006	0.65 (0.34–1.25)	0.192	0.55 (0.42–0.73)	< 0.001	0.46 (0.32–0.68)	< 0.001
	Optimal	0.47 (0.34–0.64)	< 0.001	0.45 (0.22–0.94)	0.033	0.45 (0.33–0.62)	< 0.001	0.38 (0.23–0.63)	< 0.001

Abbreviation: CVD, cardiovascular disease; HR, hazard ratio; CI, confidence interval; Ref, reference

Note: ^**#**^Model 1: univariate cox analysis; adjusting for none;

^**#**^Model 2: multivariate cox analysis; adjusting age, race, education level, PIR, HDL-C, family history of CVD, history of CVD, hypertension, and diabetes for all-cause mortality; adjusting age, race, education level, PIR, HDL-C, history of CVD, hypertension, and diabetes for CVD death.

^*^Model 1: univariate cox analysis; adjusting for none;

^*^Model 2: multivariate cox analysis; adjusting age, race, education level, PIR, LDL-C, TC, history of CVD, hypertension, and diabetes for all-cause mortality; adjusting age, education level, PIR, LDL-C, TC, history of CVD, hypertension, and diabetes for CVD death.

### Effect of overall lifestyle on the risk of all-cause mortality and CVD death in dyslipidemia patients with or without lipid-lowering therapy based on age

After adjusting race, education level, PIR, HDL-C, family history of CVD, history of CVD, hypertension, and diabetes, optimal overall lifestyle was found to be associated with the lower risk of all-cause mortality in patients without lipid-lowering therapy and with age < 65 years (HR = 0.53, 95%CI: 0.32–0.88) and age ≥ 65 years (HR = 0.57, 95%CI: 0.33–0.99). After adjusting race, education level, PIR, HDL-C, history of CVD, hypertension, and diabetes, intermediate overall lifestyle was associated with the lower risk of CVD death in patients without lipid-lowering therapy and with age < 65 years (HR = 0.34, 95%CI: 0.14–0.83). In patients with lipid-lowering therapy, better overall lifestyle was found to be associated with lower risk of all-cause mortality (intermediate: HR = 0.65, 95%CI: 0.46–0.91; optimal: HR = 0.56, 95%CI: 0.39–0.82) in age ≥ 65 years group after adjusting race, education level, PIR, LDL-C, TC, history of CVD, hypertension, and diabetes. After adjusting education level, PIR, LDL-C, TC, history of CVD, hypertension, and diabetes, the association between better overall lifestyle and lower risk of CVD death was found in patients with lipid-lowering therapy and with age ≥ 65 years (intermediate: HR = 0.52, 95%CI: 0.33–0.81; optimal: HR = 0.50, 95%CI: 0.30–0.82) (Table 4).

**Table 4:** Association between lifestyle score level and all-cause mortality or CVD death in dyslipidemia patients with or without lipid-lowering therapy based on age

Age	Overall lifestyle score	Patients without lipid-lowering therapy^#^				Patients with lipid-lowering therapy^*^			
		All-cause mortality		CVD death		All-cause mortality		CVD death	
		HR (95%CI)	*P*	HR (95%CI)	*P*	HR (95%CI)	*P*	HR (95%CI)	*P*
< 65 years	Poor	Ref		Ref		Ref		Ref	
	Intermediate	0.76 (0.53–1.09)	0.134	0.34 (0.14–0.83)	0.017	0.62 (0.35–1.08)	0.092	0.54 (0.20–1.44)	0.219
	Optimal	0.53 (0.32–0.88)	0.014	0.36 (0.09–1.41)	0.143	0.45 (0.16–1.26)	0.126	0.18 (0.03–1.25)	0.082
≥ 65 years	Poor	Ref		Ref		Ref		Ref	
	Intermediate	0.83 (0.51–1.33)	0.432	1.37 (0.51–3.64)	0.534	0.65 (0.46–0.91)	0.012	0.52 (0.33–0.81)	0.003
	Optimal	0.57 (0.33–0.99)	0.047	0.90 (0.37–2.20)	0.810	0.56 (0.39–0.82)	0.003	0.50 (0.30–0.82)	0.006

Abbreviation: CVD, cardiovascular disease; Ref: Reference, HR: hazard ratio, CI: confidence interval

Note: ^**#**^ Adjusting race, education level, PIR, HDL-C, family history of CVD, history of CVD, hypertension, and diabetes for all-cause mortality; adjusting race, education level, PIR, HDL-C, history of CVD, hypertension, and diabetes for CVD death.

^*^ Adjusting race, education level, PIR, LDL-C, TC, history of CVD, hypertension, and diabetes for all-cause mortality; adjusting education level, PIR, LDL-C, TC, history of CVD, hypertension, and diabetes for CVD death.

## Discussion

In this cohort study, 11,549 dyslipidemia patients from NHANES database were included. We found that better overall lifestyle was associated with the decreased risk of all-cause mortality and CVD death in dyslipidemia patients with or without lipid-lowering therapy. In subgroup of age ≥ 65 years, better overall lifestyle reduced the risk of all-cause mortality in patients without lipid-lowering therapy, and reduced the risk of all-cause mortality and CVD death in patients with lipid-lowering therapy. In subgroup of age < 65 years, better overall lifestyle reduced the risk of all-cause mortality and CVD death in patients without lipid-lowering therapy.

Healthy lifestyle behaviors are considered as important ways to decrease the burden of diseases [20, 21]. Keeping a healthy diet that eating more vegetables, fruit, legumes, grains, and fish, and decreasing the intake of salt, sugar, fats, and red meat prevents about 11 million deaths every year worldwide [22]. Non-smoking, alcohol restriction, and adequate physical activity respectively prevent about 7 million, 3 million, and 1 million deaths annually [22]. Combining the healthy lifestyle behaviors results in greater health benefits than the single behavior, preventing more than 60% of premature deaths [23] and extending the expected life of patients without CVD, cancer, and type 2 diabetes by 7–10 years [24]. Dyslipidemia is the main risk factor for the development of atherosclerosis, and lifestyle interventions and lipid-lowering therapy are widely used in the management of CVD [25]. Many lifestyle factors are reported to contribute to the development of dyslipidemia, including dietary habits, increased BMI, and smoking [26]. Low intensity of physical activity and short sleeping duration have also been associated with dyslipidemia [27, 28]. Some studies have reported the effect of lifestyle adjustment on the prognosis of dyslipidemia patients [4, 5]. Kokkinos et al. have reported that the increase of fitness decreased the risk of mortality in dyslipidemia patients with lipid-lowering therapy, and the risk reduced by 70% in highly fit individuals compared to least fit individuals, highlighting the importance of physical activity for dyslipidemia patients [5]. Jula et al. have reported that dietary adjustment decreased TC level by 7.6%, LDL-C level by 10.8%, HDL-C level by 4.9%, and apolipoprotein B level by 5.7% in patients with hypercholesterolemia [4]. In this study, we found that overall lifestyle assessed by Mediterranean diet score, physical activity, smoking status, sleep duration, and BMI was associated with prognosis of dyslipidemia patients. Better overall lifestyle decreased the risk of all-cause mortality and CVD death in patients with or without lipid-lowering therapy. Our findings highlighted the importance of overall lifestyle adjustment in the improvement of poor prognosis for dyslipidemia patients whether receiving lipid-lowering treatment or not.

Age has been reported to be associated with dyslipidemia [29]. Herein, we explored the effect of overall lifestyle on the all-cause mortality and CVD death based on ages. For dyslipidemia patients with age ≥ 65 years, whether taking lipid-lowering treatment or not, better overall lifestyle contributes to reduce the risk of all-cause mortality. Molanorouzi et al. have found that older individuals exhibited greater concerns about health outcomes, which motivated them to adjust lifestyle for better physical and psychological health [30]. Better overall lifestyle was associated with the reduced risk of CVD death in patients with lipid-lowering treatment, while no significance was found in patients without lipid-lowering treatment, indicating that better overall lifestyle may have potential benefits to reduce the risk of CVD death and was especially important for the elderly receiving lipid-lowering treatment. For dyslipidemia patients with age < 65 years, better overall lifestyle was associated with the reduced risk of all-cause mortality and CVD death in the individuals without lipid-lowering therapy. Our findings indicated that a good overall lifestyle may help improve the prognosis of younger dyslipidemia patients not receiving lipid-lowering treatments.

This is a cohort study including a larger sample size to explore the effect of overall lifestyle on the prognosis of dyslipidemia patients, which provides evidence for the importance of lifestyle adjustment in the management of dyslipidemia. However, there are some limitations in this study. First, this is a retrospective study that selective bias is inevitable. Second, there is no information on the dosage and frequency of lipid-lowering medications due to the limitation of database; therefore, we cannot assess the effect of overall lifestyle on the prognosis based on dosage and frequency of lipid-lowering medication, which needs to be further explored in future studies. Third, although the five lifestyle factors included in the overall lifestyle score are the main components reported in previous studies, they may not represent all aspects of the lifestyle. Future studies should include more lifestyle factors to verify our findings.

## Conclusion

In this study, we found that better overall lifestyle was associated with the reduced risk of all-cause mortality and CVD death in dyslipidemia patients with or without lipid-lowering therapy, indicating that adherence to a favorable lifestyle may have great benefits for the management of dyslipidemia whether receiving lipid-lowering treatment or not.

### Electronic supplementary material

Below is the link to the electronic supplementary material.

Additional File 1: table s1 - s4

## Data Availability

The datasets generated and/or analyzed during the current study are available in the NHANES database, https://wwwn.cdc.gov/nchs/nhanes/.

## List of abbreviations

TC: Total cholesterol
LDL-C: Low density lipoprotein-cholesterol
TG: Triglycerides
HDL-C: High-density lipoprotein cholesterol
CVD: Cardiovascular disease
AHA: American Heart Association
BMI: Body mass index
NHANES: National Health and Nutrition Examination Survey
NCHS: National Center for Health Statistics
CDC: Centers for Disease Control and Prevention
ERB: Ethics Review Board
PIR: Poverty income ratio
HR: Hazard ratio
