# Supplementary material for: Effect of overall lifestyle on the all-cause mortality and cardiovascular disease death in dyslipidemia patients with or without lipid-lowering therapy: a cohort study
Source: BMC Cardiovasc Disord. 2023 Sep 4;23:438. doi: 10.1186/s12872-023-03450-1 (PMC10476373; doi:10.1186/s12872-023-03450-1)
Supplement: Supplementary file 1 — Additional File 1: table s1 - s4 [file 12872_2023_3450_MOESM1_ESM.docx]

**Supplementary table S1 Sensitivity analysis for multiple imputation**

| **Variables** | **Total (n = 23098)** | **Before imputation (n = 11549)** | **After imputation (n = 11549)** | **Statistics** | ***P*** |
| --- | --- | --- | --- | --- | --- |
| Age, year, Mean ± SD | 53.54 ± 17.08 | 53.54 ± 17.08 | 53.54 ± 17.08 | t = -0.00 | 1.000 |
| Gender, n (%) |  |  |  | χ^2^ = 0.000 | 1.000 |
| Male | 11666 (50.51) | 5833 (50.51) | 5833 (50.51) |  |  |
| Female | 11432 (49.49) | 5716 (49.49) | 5716 (49.49) |  |  |
| Race, n (%) |  |  |  | χ^2^ = 0.000 | 1.000 |
| Mexican American | 3632 (15.72) | 1816 (15.72) | 1816 (15.72) |  |  |
| Other Hispanic | 2416 (10.46) | 1208 (10.46) | 1208 (10.46) |  |  |
| Non-Hispanic white | 10246 (44.36) | 5123 (44.36) | 5123 (44.36) |  |  |
| Non-Hispanic black | 4330 (18.75) | 2165 (18.75) | 2165 (18.75) |  |  |
| Other Race-including multi-racial | 2474 (10.71) | 1237 (10.71) | 1237 (10.71) |  |  |
| Education level, n (%) |  |  |  | χ^2^ = 0.005 | 1.000 |
| Less than 9^th^ grade | 2757 (11.94) | 1377 (11.93) | 1380 (11.95) |  |  |
| 9-11^th^ grade (including 12^th^ grade with no diploma) | 3465 (15.01) | 1732 (15.01) | 1733 (15.01) |  |  |
| High school graduate/ GED or equivalent | 5438 (23.55) | 2716 (23.54) | 2722 (23.57) |  |  |
| More than high school | 11427 (49.50) | 5713 (49.51) | 5714 (49.48) |  |  |
| PIR, %, M (Q_1_, Q_3_) | 2.14 (1.15, 4.10) | 2.14 (1.15, 4.12) | 2.14 (1.16, 4.08) | Z = -1.739 | 0.082 |
| Triglyceride, mmol/L, M (Q_1_, Q_3_) | 1.36 (0.96, 1.94) | 1.36 (0.96, 1.94) | 1.36 (0.96, 1.94) | Z = 0.000 | 1.000 |
| LDL-C, mmol/L, M (Q_1_, Q_3_) | 3.15 ± 0.98 | 3.15 ± 0.98 | 3.15 ± 0.98 | t = -0.00 | 1.000 |
| TC, mmol/L, Mean ± SD | 5.24 ± 1.11 | 5.24 ± 1.11 | 5.24 ± 1.11 | t = -0.00 | 1.000 |
| HDL-C, mmol/L, Mean ± SD | 1.38 ± 0.44 | 1.38 ± 0.44 | 1.38 ± 0.44 | t = 0.00 | 1.000 |
| History of CVD, n (%) |  |  |  | χ^2^ = 0.000 | 0.997 |
| Yes | 3330 (14.51) | 1654 (14.51) | 1676 (14.51) |  |  |
| No | 19615 (85.49) | 9742 (85.49) | 9873 (85.49) |  |  |
| Family history of CVD, n (%) |  |  |  | χ^2^ = 0.000 | 1.000 |
| Yes | 3080 (13.33) | 1540 (13.33) | 1540 (13.33) |  |  |
| No | 19236 (83.28) | 9618 (83.28) | 9618 (83.28) |  |  |
| Unknown | 782 (3.39) | 391 (3.39) | 391 (3.39) |  |  |
| Hypertension, n (%) |  |  |  | χ^2^ = 0.000 | 1.000 |
| Yes | 12942 (56.03) | 6471 (56.03) | 6471 (56.03) |  |  |
| No | 10156 (43.97) | 5078 (43.97) | 5078 (43.97) |  |  |
| Diabetes, n (%) |  |  |  | χ^2^ = 0.000 | 1.000 |
| Yes | 6018 (26.05) | 3009 (26.05) | 3009 (26.05) |  |  |
| No | 17080 (73.95) | 8540 (73.95) | 8540 (73.95) |  |  |
| BMI, kg/m^2^, Mean ± SD | 29.71 ± 6.75 | 29.71 ± 6.75 | 29.71 ± 6.75 | t = 0.00 | 1.000 |
| Smoking status, n (%) |  |  |  | χ^2^ = 0.000 | 1.000 |
| Poor | 4608 (19.95) | 2304 (19.95) | 2304 (19.95) |  |  |
| Intermediate | 6252 (27.07) | 3126 (27.07) | 3126 (27.07) |  |  |
| Optimal | 12238 (52.98) | 6119 (52.98) | 6119 (52.98) |  |  |
| Sleep duration, n (%) |  |  |  | χ^2^ = 0.000 | 1.000 |
| Poor | 1634 (7.07) | 817 (7.07) | 817 (7.07) |  |  |
| Intermediate | 4996 (21.63) | 2498 (21.63) | 2498 (21.63) |  |  |
| Optimal | 16468 (71.30) | 8234 (71.30) | 8234 (71.30) |  |  |
| BMI category, n (%) |  |  |  | χ^2^ = 0.000 | 1.000 |
| Poor | 9432 (40.83) | 4716 (40.83) | 4716 (40.83) |  |  |
| Intermediate | 8370 (36.24) | 4185 (36.24) | 4185 (36.24) |  |  |
| Optimal | 5296 (22.93) | 2648 (22.93) | 2648 (22.93) |  |  |
| Mediterranean diet score, n (%) |  |  |  | χ^2^ = 0.000 | 1.000 |
| Poor | 17712 (76.68) | 8856 (76.68) | 8856 (76.68) |  |  |
| Intermediate | 5258 (22.76) | 2629 (22.76) | 2629 (22.76) |  |  |
| Optimal | 128 (0.55) | 64 (0.55) | 64 (0.55) |  |  |
| Physical activity level, n (%) |  |  |  | χ^2^ = 0.000 | 1.000 |
| Low | 12500 (54.12) | 6250 (54.12) | 6250 (54.12) |  |  |
| High | 10598 (45.88) | 5299 (45.88) | 5299 (45.88) |  |  |
| Lipid-lowering therapy, n (%) |  |  |  | χ^2^ = 0.000 | 1.000 |
| No | 15672 (67.85) | 7836 (67.85) | 7836 (67.85) |  |  |
| Yes | 7426 (32.15) | 3713 (32.15) | 3713 (32.15) |  |  |
| Follow-up, month, M (Q_1_, Q_3_) | 89.00 (49.00, 130.00) | 89.00 (49.00, 130.00) | 89.00 (49.00, 130.00) | Z = 0.000 | 1.000 |
| CVD mortality, n (%) |  |  |  | χ^2^ = 0.000 | 1.000 |
| Survival | 20266 (87.74) | 10133 (87.74) | 10133 (87.74) |  |  |
| CVD death | 918 (3.97) | 459 (3.97) | 459 (3.97) |  |  |
| Other death | 1914 (8.29) | 957 (8.29) | 957 (8.29) |  |  |
| All-cause mortality, n (%) |  |  |  | χ^2^ = 0.000 | 1.000 |
| Survival | 20266 (87.74) | 10133 (87.74) | 10133 (87.74) |  |  |
| Death | 2832 (12.26) | 1416 (12.26) | 1416 (12.26) |  |  |
| Lifestyle score level, n (%) |  |  |  | χ^2^ = 0.000 | 1.000 |
| Poor | 2284 (9.89) | 1142 (9.89) | 1142 (9.89) |  |  |
| Intermediate | 14878 (64.41) | 7439 (64.41) | 7439 (64.41) |  |  |
| Optimal | 5936 (25.70) | 2968 (25.70) | 2968 (25.70) |  |  |

Abbreviation: Mean ± SD, mean ± standard deviation; GED, General Educational Development; PIR, poverty-income ratio; LDL-C, low-density lipoprotein cholesterol; TC, total cholesterol; HDL-C, high-density lipoprotein cholesterol; CVD, cardiovascular disease; BMI, body mass index.

χ^2^: chi-square test; F: variance analysis.

**Supplementary table S2** **Characteristics of patients without lipid-lowering therapy**

| **Variables** | **Total (n=7836)** | **Overall lifestyle score** | | | **Statistics** | *P* |
| --- | --- | --- | --- | --- | --- | --- |
|  |  | **Poor (n = 685)** | **Intermediate (n = 4823)** | **Optimal (n = 2328)** |  |  |
| Age, year, Mean ± SD | 47.00 (35.00, 60.00) | 47.00 (36.00, 60.00) | 47.00 (35.00, 60.00) | 47.00 (35.00, 60.00) | χ^2^ = 0.054 | 0.973^#^ |
| Gender, n (%) |  |  |  |  | χ^2^ = 2.848 | 0.241 |
| Male | 3903 (49.81) | 339 (49.49) | 2437 (50.53) | 1127 (48.41) |  |  |
| Female | 3933 (50.19) | 346 (50.51) | 2386 (49.47) | 1201 (51.59) |  |  |
| Race, n (%) |  |  |  |  | χ^2^ = 253.339 | < 0.001 |
| Mexican American | 1388 (17.71) | 90 (13.14) | 890 (18.45) | 408 (17.53) |  |  |
| Other Hispanic | 858 (10.95) | 71 (10.36) | 532 (11.03) | 255 (10.95) |  |  |
| Non-Hispanic white | 3296 (42.06) | 299 (43.65) | 2052 (42.55) | 945 (40.59) |  |  |
| Non-Hispanic black | 1414 (18.04) | 177 (25.84) | 953 (19.76) | 284 (12.20) |  |  |
| Other race-including multi-racial | 880 (11.23) | 48 (7.01) | 396 (8.21) | 436 (18.73) |  |  |
| Education level, n (%) |  |  |  |  | χ^2^ = 228.137 | < 0.001 |
| Less than 9^th^ grade | 866 (11.05) | 81 (11.82) | 572 (11.86) | 213 (9.15) |  |  |
| 9-11^th^ grade (Includes 12^th^ grade with no diploma) | 1186 (15.14) | 166 (24.23) | 791 (16.40) | 229 (9.84) |  |  |
| High school graduate/ GED or equivalent | 1796 (22.92) | 198 (28.91) | 1159 (24.03) | 439 (18.86) |  |  |
| More than high school | 3988 (50.89) | 240 (35.04) | 2301 (47.71) | 1447 (62.16) |  |  |
| PIR, %, M (Q_1_, Q_3_) | 2.10 (1.12, 4.07) | 1.36 (0.87, 2.59) | 2.00 (1.08, 3.98) | 2.98 (1.44, 5.00) | χ^2^ = 306.473 | < 0.001^#^ |
| Triglyceride, mmol/L, M (Q_1_, Q_3_) | 1.39 (0.97, 2.00) | 1.61 (1.11, 2.18) | 1.47 (1.03, 2.09) | 1.22 (0.86, 1.76) | χ^2^ = 224.038 | < 0.001^#^ |
| LDL-C, mmol/L, M (Q_1_, Q_3_) | 3.43 ± 0.88 | 3.37 ± 0.93 | 3.44 ± 0.91 | 3.45 ± 0.82 | F = 2.591 | 0.075 |
| TC, mmol/L, Mean ± SD | 5.54 ± 1.00 | 5.39 ± 1.04 | 5.53 ± 1.02 | 5.60 ± 0.94 | F = 12.641 | < 0.001 |
| HDL-C, mmol/L, Mean ± SD | 1.29 (1.03, 1.63) | 1.14 (0.93, 1.42) | 1.24 (1.01, 1.55) | 1.42 (1.14, 1.84) | χ^2^ = 350.638 | < 0.001^#^ |
| History of CVD, n (%) |  |  |  |  | χ^2^ = 103.209 | < 0.001 |
| Yes | 497 (6.34) | 97 (14.16) | 319 (6.61) | 81 (3.48) |  |  |
| No | 7339 (93.66) | 588 (85.84) | 4504 (93.39) | 2247 (96.52) |  |  |
| Family history of CVD, n (%) |  |  |  |  | χ^2^ = 52.456 | < 0.001 |
| Yes | 933 (11.91) | 122 (17.81) | 612 (12.69) | 199 (8.55) |  |  |
| No | 6641 (84.75) | 541 (78.98) | 4042 (83.81) | 2058 (88.40) |  |  |
| Unknown | 262 (3.34) | 22 (3.21) | 169 (3.50) | 71 (3.05) |  |  |
| Hypertension, n (%) |  |  |  |  | χ^2^ = 148.486 | < 0.001 |
| Yes | 3482 (44.44) | 380 (55.47) | 2300 (47.69) | 802 (34.45) |  |  |
| No | 4354 (55.56) | 305 (44.53) | 2523 (52.31) | 1526 (65.55) |  |  |
| Diabetes, n (%) |  |  |  |  | χ^2^ = 111.578 | < 0.001 |
| Yes | 1185 (15.12) | 163 (23.80) | 806 (16.71) | 216 (9.28) |  |  |
| No | 6651 (84.88) | 522 (76.20) | 4017 (83.29) | 2112 (90.72) |  |  |
| BMI, kg/m^2^, Mean ± SD | 29.38 ± 6.68 | 33.84 ± 6.88 | 30.62 ± 6.85 | 25.49 ± 3.89 | F = 748.899 | < 0.001 |
| Smoking status, n (%) |  |  |  |  | χ^2^ = 2161.985 | < 0.001 |
| Poor | 1738 (22.18) | 512 (74.74) | 1195 (24.78) | 31 (1.33) |  |  |
| Intermediate | 1765 (22.52) | 126 (18.39) | 1317 (27.31) | 322 (13.83) |  |  |
| Optimal | 4333 (55.30) | 47 (6.86) | 2311 (47.92) | 1975 (84.84) |  |  |
| Sleep duration, n (%) |  |  |  |  | χ^2^ = 1813.654 | < 0.001 |
| Poor | 529 (6.75) | 264 (38.54) | 263 (5.45) | 2 (0.09) |  |  |
| Intermediate | 1625 (20.74) | 254 (37.08) | 1173 (24.32) | 198 (8.51) |  |  |
| Optimal | 5682 (72.51) | 167 (24.38) | 3387 (70.23) | 2128 (91.41) |  |  |
| BMI category, n (%) |  |  |  |  | χ^2^ = 1926.050 | < 0.001 |
| Poor | 2998 (38.26) | 525 (76.64) | 2302 (47.73) | 171 (7.35) |  |  |
| Intermediate | 2911 (37.15) | 135 (19.71) | 1779 (36.89) | 997 (42.83) |  |  |
| Optimal | 1927 (24.59) | 25 (3.65) | 742 (15.38) | 1160 (49.83) |  |  |
| Mediterranean diet score, n (%) |  |  |  |  | χ^2^ = 1353.827 | < 0.001 |
| Poor | 6070 (77.46) | 655 (95.62) | 3934 (81.57) | 1020 (43.81) |  |  |
| Intermediate | 1723 (21.99) | 30 (4.38) | 886 (18.37) | 1250 (53.69) |  |  |
| Optimal | 43 (0.55) | 0 (0.00) | 3 (0.06) | 58 (2.49) |  |  |
| Physical activity level, n (%) |  |  |  |  | χ^2^ = 723.732 | < 0.001 |
| Low | 3821 (48.76) | 540 (78.83) | 2634 (54.61) | 647 (27.79) |  |  |
| High | 4015 (51.24) | 145 (21.17) | 2189 (45.39) | 1681 (72.21) |  |  |
| Follow-up, M (Q_1_, Q_3_) | 95.00 (53.00, 134.00) | 86.00 (46.00, 130.00) | 95.00 (53.00, 135.00) | 95.00 (58.00, 134.00) | χ^2^ = 9.686 | 0.008^#^ |
| CVD mortality, n (%) |  |  |  |  | χ^2^ = 28.971 | < 0.001 |
| Survival | 7210 (92.01) | 604 (88.18) | 4417 (91.58) | 2189 (94.03) |  |  |
| CVD death | 178 (2.27) | 20 (2.92) | 120 (2.49) | 38 (1.63) |  |  |
| Other death | 448 (5.72) | 61 (8.91) | 286 (5.93) | 101 (4.34) |  |  |
| All-cause mortality, n (%) |  |  |  |  | χ^2^ = 27.819 | < 0.001 |
| Survival | 7210 (92.01) | 604 (88.18) | 4417 (91.58) | 2189 (94.03) |  |  |
| Death | 626 (7.99) | 81 (11.82) | 406 (8.42) | 139 (5.97) |  |  |

Abbreviation: Mean ± SD, mean ± standard deviation; GED, General Educational Development; PIR, poverty-income ratio; LDL-C, low-density lipoprotein cholesterol; TC, total cholesterol; HDL-C, high-density lipoprotein cholesterol; CVD, cardiovascular disease; BMI, body mass index.

χ^2^: chi-square test; F: variance analysis; #: rank sum test.

**Supplementary table S3** **Characteristics of patients with lipid-lowering therapy**

| **Variables** | **Total (n=3713)** | **Overall lifestyle score** | | | **Statistics** | ***P*** |
| --- | --- | --- | --- | --- | --- | --- |
|  |  | **Poor (n = 424)** | **Intermediate (n = 2469)** | **Optimal (n = 820)** |  |  |
| Age, year, Mean ± SD | 65.45 ± 11.38 | 62.39 ± 11.64 | 66.01 ± 11.15 | 65.35 ± 11.69 | F = 18.594 | < 0.001 |
| Gender, n (%) |  |  |  |  | χ^2^ = 2.219 | 0.330 |
| Male | 1930 (51.98) | 227 (53.54) | 1262 (51.11) | 441 (53.78) |  |  |
| Female | 1783 (48.02) | 197 (46.46) | 1207 (48.89) | 379 (46.22) |  |  |
| Race, n (%) |  |  |  |  | χ^2^ = 163.992 | < 0.001 |
| Mexican American | 428 (11.53) | 42 (9.91) | 307 (12.43) | 79 (9.63) |  |  |
| Other Hispanic | 350 (9.43) | 34 (8.02) | 247 (10.00) | 69 (8.41) |  |  |
| Non-Hispanic white | 1827 (49.21) | 196 (46.23) | 1239 (50.18) | 392 (47.80) |  |  |
| Non-Hispanic black | 751 (20.23) | 128 (30.19) | 507 (20.53) | 116 (14.15) |  |  |
| Other race-including multi-racial | 357 (9.61) | 24 (5.66) | 169 (6.84) | 164 (20.00) |  |  |
| Education level, n (%) |  |  |  |  | χ^2^ = 85.503 | < 0.001 |
| Less than 9^th^ grade | 514 (13.84) | 65 (15.33) | 364 (14.74) | 85 (10.37) |  |  |
| 9-11^th^ grade (including 12^th^ grade with no diploma) | 547 (14.73) | 92 (21.70) | 377 (15.27) | 78 (9.51) |  |  |
| High school graduate/ GED or equivalent | 926 (24.94) | 124 (29.25) | 622 (25.19) | 180 (21.95) |  |  |
| More than high school | 1726 (46.49) | 143 (33.73) | 1106 (44.80) | 477 (58.17) |  |  |
| PIR, %, M (Q_1_, Q_3_) | 2.20 (1.23, 4.10) | 1.65 (1.00, 3.00) | 2.14 (1.22, 4.00) | 3.14 (1.73, 5.00) | χ^2^ = 135.686 | < 0.001^#^ |
| Triglyceride, mmol/L, M (Q_1_, Q_3_) | 1.29 (0.93, 1.83) | 1.46 (1.04, 2.04) | 1.34 (0.95, 1.87) | 1.13 (0.78, 1.58) | χ^2^ = 93.526 | < 0.001^#^ |
| LDL-C, mmol/L, M (Q_1_, Q_3_) | 2.43 (1.94, 3.03) | 2.41 (1.88, 3.12) | 2.43 (1.94, 3.03) | 2.48 (1.97, 3.03) | χ^2^ = 0.307 | 0.858^#^ |
| TC, mmol/L, Mean ± SD | 4.60 ± 1.05 | 4.60 ± 1.12 | 4.60 ± 1.04 | 4.61 ± 1.04 | F = 0.019 | 0.981 |
| HDL-C, mmol/L, Mean ± SD | 1.37 ± 0.39 | 1.26 ± 0.37 | 1.36 ± 0.39 | 1.46 ± 0.39 | F = 40.357 | < 0.001 |
| History of CVD, n (%) |  |  |  |  | χ^2^ = 41.195 | < 0.001 |
| Yes | 1179 (31.75) | 180 (42.45) | 796 (32.24) | 203 (24.76) |  |  |
| No | 2534 (68.25) | 244 (57.55) | 1673 (67.76) | 617 (75.24) |  |  |
| Family history of CVD, n (%) |  |  |  |  | χ^2^ = 25.837 | < 0.001 |
| Yes | 607 (16.35) | 104 (24.53) | 387 (15.67) | 116 (14.15) |  |  |
| No | 2977 (80.18) | 305 (71.93) | 1992 (80.68) | 680 (82.93) |  |  |
| Unknown | 129 (3.47) | 15 (3.54) | 90 (3.65) | 24 (2.93) |  |  |
| Hypertension, n (%) |  |  |  |  | χ^2^ = 50.604 | < 0.001 |
| Yes | 2989 (80.50) | 355 (83.73) | 2045 (82.83) | 589 (71.83) |  |  |
| No | 724 (19.50) | 69 (16.27) | 424 (17.17) | 231 (28.17) |  |  |
| Diabetes, n (%) |  |  |  |  | χ^2^ = 63.309 | < 0.001 |
| Yes | 1824 (49.12) | 258 (60.85) | 1251 (50.67) | 315 (38.41) |  |  |
| No | 1889 (50.88) | 166 (39.15) | 1218 (49.33) | 505 (61.59) |  |  |
| BMI, kg/m^2^, Mean ± SD | 30.43 ± 6.83 | 34.60 ± 7.19 | 31.18 ± 6.66 | 26.00 ± 4.53 | F = 311.781 | < 0.001 |
| Smoking status, n (%) |  |  |  |  | χ^2^ = 954.578 | < 0.001 |
| Poor | 566 (15.24) | 233 (54.95) | 324 (13.12) | 9 (1.10) |  |  |
| Intermediate | 1361 (36.65) | 154 (36.32) | 1048 (42.45) | 159 (19.39) |  |  |
| Optimal | 1786 (48.10) | 37 (8.73) | 1097 (44.43) | 652 (79.51) |  |  |
| Sleep duration, n (%) |  |  |  |  | χ^2^ = 992.130 | < 0.001 |
| Poor | 288 (7.76) | 170 (40.09) | 116 (4.70) | 2 (0.24) |  |  |
| Intermediate | 873 (23.51) | 161 (37.97) | 648 (26.25) | 64 (7.80) |  |  |
| Optimal | 2552 (68.73) | 93 (21.93) | 1705 (69.06) | 754 (91.95) |  |  |
| BMI category, n (%) |  |  |  |  | χ^2^ = 980.563 | < 0.001 |
| Poor | 1718 (46.27) | 354 (83.49) | 1294 (52.41) | 70 (8.54) |  |  |
| Intermediate | 1274 (34.31) | 60 (14.15) | 872 (35.32) | 342 (41.71) |  |  |
| Optimal | 721 (19.42) | 10 (2.36) | 303 (12.27) | 408 (49.76) |  |  |
| Mediterranean diet score, n (%) |  |  |  |  | χ^2^ = 598.561 | < 0.001 |
| Poor | 2786 (75.03) | 406 (95.75) | 1875 (75.94) | 312 (38.05) |  |  |
| Intermediate | 906 (24.40) | 18 (4.25) | 589 (23.86) | 483 (58.90) |  |  |
| Optimal | 21 (0.57) | 0 (0.00) | 5 (0.20) | 25 (3.05) |  |  |
| Physical activity level, n (%) |  |  |  |  | χ^2^ = 503.012 | < 0.001 |
| Low | 2429 (65.42) | 391 (92.22) | 1751 (70.92) | 287 (35.00) |  |  |
| High | 1284 (34.58) | 33 (7.78) | 718 (29.08) | 533 (65.00) |  |  |
| Follow-up, month, M (Q_1_, Q_3_) | 79.00 (42.00, 120.00) | 72.00 (33.00, 113.00) | 77.00 (42.00, 120.00) | 85.00 (50.00, 122.00) | χ^2^ = 17.604 | < 0.001^#^ |
| CVD mortality, n (%) |  |  |  |  | χ^2^ = 16.491 | 0.002 |
| Survival | 2923 (78.72) | 313 (73.82) | 1928 (78.09) | 682 (83.17) |  |  |
| CVD death | 281 (7.57) | 41 (9.67) | 191 (7.74) | 49 (5.98) |  |  |
| Other death | 509 (13.71) | 70 (16.51) | 350 (14.18) | 89 (10.85) |  |  |
| All-cause mortality, n (%) |  |  |  |  | χ^2^ = 16.362 | < 0.001 |
| Survival | 2923 (78.72) | 313 (73.82) | 1928 (78.09) | 682 (83.17) |  |  |
| Death | 790 (21.28) | 111 (26.18) | 541 (21.91) | 138 (16.83) |  |  |

Abbreviation: Mean ± SD, mean ± standard deviation; GED, General Educational Development; PIR, poverty-income ratio; LDL-C, low-density lipoprotein cholesterol; TC, total cholesterol; HDL-C, high-density lipoprotein cholesterol; CVD, cardiovascular disease; BMI, body mass index.

χ^2^: chi-square test; F: variance analysis; #: rank sum test.

**Supplementary table S4 Sensitivity analysis for the association between overall lifestyle score (removing BMI) and all-cause mortality and CVD death**

| **Model** | **Overall lifestyle score** | **Patients without lipid-lowering therapy^#^** | | | | **Patients with lipid-lowering therapy^*^** | | | |
| --- | --- | --- | --- | --- | --- | --- | --- | --- | --- |
|  |  | **All-cause mortality** | | **CVD death** | | **All-cause mortality** | | **CVD death** | |
|  |  | **HR (95%CI)** | ***P*** | **HR (95%CI)** | ***P*** | **HR (95%CI)** | ***P*** | **HR (95%CI)** | ***P*** |
| Model 1 | Poor | Ref |  | Ref |  | Ref |  | Ref |  |
|  | Intermediate | 0.67 (0.49-0.91) | 0.011 | 0.68 (0.31-1.51) | 0.348 | 0.65 (0.50-0.86) | 0.003 | 0.62 (0.36-1.07) | 0.088 |
|  | Optimal | 0.27 (0.19-0.38) | < 0.001 | 0.36 (0.14-0.87) | 0.024 | 0.26 (0.19-0.35) | < 0.001 | 0.28 (0.15-0.52) | < 0.001 |
| Model 2 | Poor | Ref |  | Ref |  | Ref |  | Ref |  |
|  | Intermediate | 0.65 (0.47-0.89) | 0.008 | 0.57 (0.24-1.34) | 0.194 | 0.50 (0.38-0.65) | < 0.001 | 0.43 (0.24-0.77) | < 0.001 |
|  | Optimal | 0.33 (0.24-0.46) | < 0.001 | 0.42 (0.15-1.14) | 0.087 | 0.29 (0.22-0.38) | < 0.001 | 0.32 (0.16-0.61) | < 0.001 |

Abbreviation: BMI, body mass index; CVD, cardiovascular disease; Ref: Reference, HR: hazard ratio, CI: confidence interval.

Note: **^#^** Adjusting age, race, education level, PIR, HDL-C, family history of CVD, history of CVD, hypertension, diabetes, and BMI for all-cause mortality; adjusting age, race, education level, PIR, HDL-C, history of CVD, hypertension, diabetes, and BMI for CVD death.

**^*^** Adjusting age, race, education level, PIR, TC, LDL-C, history of CVD, hypertension, diabetes, and BMI for all-cause mortality; adjusting age, education level, PIR, TC, LDL-C, history of CVD, hypertension, diabetes, and BMI for CVD death.
